# Pterostilbene Eliminates MRSA Independent of Metabolic State and Effectively Prevents Biofilm Formation in Milk Matrices

**DOI:** 10.3390/foods14183236

**Published:** 2025-09-18

**Authors:** Xiaoyong Huang, Huiting Yang, Chenli Wang, Shiqi Yan, Xiaomin Ren, Zilong Sun

**Affiliations:** College of Veterinary Medicine, Shanxi Agricultural University, Taiyuan 030031, China; yanghuiting0321@163.com (H.Y.); wcl0070@yeah.net (C.W.); yanshiqi0504@163.com (S.Y.); renxiaomin1225@163.com (X.R.)

**Keywords:** PT, MRSA, membrane permeability, phospholipids, biofilm

## Abstract

The relentless spread of antimicrobial resistance poses a severe threat to global public health, food safety, and environmental security. Natural products with potent antimicrobial activity offer prospective substitutes for traditional antibiotics and chemical preservatives. Here, we demonstrate that pterostilbene (PT), a natural dietary compound, exhibits rapid lytic activity against methicillin-resistant *Staphylococcus aureus* (MRSA). PT displayed metabolism-independent bactericidal effects, effectively eradicating dormant persister cells within one hour, though its activity was partially attenuated under anaerobic conditions. Mechanistically, PT disrupted membrane integrity by increasing permeability, dissipating membrane potential, depleting cellular ATP, and suppressing reactive oxygen species (ROS) generation. Its efficacy was modulated by membrane phospholipid composition, with phosphatidylglycerol (PG) and cardiolipin (CL) critically influencing antimicrobial potency. Crucially, PT robustly inhibited MRSA biofilm formation in milk. These findings highlight PT’s potential as a structurally stable, natural antimicrobial for controlling resilient MRSA, particularly against biofilm-associated and persister subpopulations in food systems.

## 1. Introduction

The relentless evolution of antimicrobial resistance (AMR) has propelled methicillin-resistant *Staphylococcus aureus* (MRSA) into a global priority pathogen posing a widespread threat to food safety [1]. This Gram-positive pathogen is ubiquitous in nature and readily contaminates diverse food matrices, including milk, meat, eggs, and ready-to-eat products, leading to food poisoning and several life-threatening infectious diseases [2,3]. Alarmingly, 90.30% of 165 *S. aureus* isolates recovered from 95 food poisoning episodes (2006–2019) carried high-risk toxin genes, underscoring its public health burden [4]. According to statistics, the number of deaths linked to and caused by MRSA increased the most over the past three decades globally [5]. Thus, the dwindling efficacy of conventional antibiotics and preservatives necessitates urgent exploration of antimicrobials with novel mechanisms.

Natural products with high antimicrobial activity have gained considerable attention due to their potential to serve as alternatives to conventional antibiotics and chemical preservatives [6,7]. These compounds are frequently obtained from multiple plants, animals, and microbial sources and exhibit a wide variety of chemical structures and modes of action against resistant strains mitigating the risk of further resistance development [8]. Moreover, the rising consumer demand for eco-friendly products [9] and concerns over synthetic additives have also pushed the research direction for natural compounds as viable alternatives for use in various applications, including food and livestock farming. Nowadays, many researchers are focusing on isolating, identifying, and characterizing novel natural antimicrobials from diverse sources. This has led to the discovery of numerous compounds with promising antimicrobial activities. For example, plant-derived flavonoids α-mangostin (AMG) and isobavachalcone (IBC) [10], as well as marine-derived new peptaibols [11], exhibit rapid bactericidal activity against multidrug resistant pathogens. These compounds offer a promising approach to developing novel antimicrobial agents that are both effective and environmentally friendly.

PT (3,5-dimethoxy-4′-hydroxy-trans-stilbene), a naturally abundant stilbenoid found in sources such as *Pterocarpus marsupium* and blueberries, represents a highly promising nutraceutical analog of resveratrol [12]. Beyond its well-documented efficacy in inflammation modulation and cancer chemoprevention [13,14,15], effects partially through modulating the gut microbiota [16,17], PT also demonstrates potent and broad-spectrum antimicrobial activity. This activity involves notable inhibitory actions against a wide variety of pathogens, including Gram-positive bacteria [18,19], Gram-negative bacteria [20], and various fungal species [21]. Meanwhile, PT restores bacterial susceptibility to antibiotics like carbapenem and Polymyxin B in resistant strains [22,23]. Furthermore, PT has shown promise in disrupting bacterial biofilms [19,24], a major factor in chronic infections and antimicrobial resistance. However, despite these compelling observations of its antimicrobial potency, the precise molecular mechanisms underpinning the antibacterial actions of PT remain inadequately elucidated.

Here, we explored the antibacterial modes of PT against MRSA. We found its rapid, metabolism-independent lytic activity of its bactericidal action including efficacy against MRSA persister cells. Furthermore, we characterized the role of membrane phospholipids (phosphatidylglycerol and cardiolipin) in its antibacterial effects while elucidating impacts on the generation of ROS, membrane potential dissipation, ATP depletion, and membrane permeability. Our findings demonstrate that PT represents a structurally stable, multimodal anti-MRSA agent with activity across metabolic states, offering a promising scaffold for combating multidrug-resistant *staphylococcal* infections.

## 2. Methods

### 2.1. Compounds and Bacterial Strains

PT (C_16_H_16_O_3_, CAS:537-42-8, purity ≥ 97%) was purchased from Macklin Biochemical Co., Ltd., Shanghai, China. PT was dissolved in dimethyl sulfoxide (DMSO) to prepare a 10 mg/mL stock solution, which was stored at −20 °C. Working solutions were diluted with no cation-adjusted Mueller–Hinton Broth (MHB, SanYao, Beijing, China) or phosphate-buffered saline (PBS; pH 7.4) just prior to use. The final concentration of DMSO was always kept below 1% (*v*/*v*) in all experimental treatments. A vehicle control containing the same highest percentage of DMSO was included in all assays, and it was confirmed to have no detectable inhibitory effect on bacterial growth compared to a DMSO-free control. PBS used for washing and dilution contained 137 mM NaCl, 10 mM Na_2_HPO_4_, and 1.8 mM KH_2_PO_4_. The MRSA strain T144, which demonstrates high-level resistance to multiple antimicrobial classes, was selected for the antibacterial assay. It exhibited elevated minimum inhibitory concentrations (MICs) to fluoroquinolones (ciprofloxacin, MIC > 128 mg/L), cephalosporins (ceftiofur, MIC > 128 mg/L), sulfonamides (sulfamethoxazole, MIC > 128 mg/L), macrolides (erythromycin, MIC > 128 mg/L), and aminoglycosides (gentamicin, MIC = 64 mg/L). Additionally, two animal-derived MRSA strains MRSA CAU183 and CAU184 were also included in this study. The further details about bacterial strains can be found in ref. [10].

### 2.2. Growth Curve Analysis

Bacterial growth kinetics under sub-inhibitory PT concentrations were assessed in MHB. Mid-log cultures were diluted to ~10^6^ CFU/mL in MHB containing PT (0, 4, 8, and 16 mg/L). Under anaerobic growth conditions, wells were overlaid with mineral oil, following a previous study [25]. Optical absorbance at 600 nm (OD_600_) was recorded for 24 h using a microplate reader (Tecan Infinite E PLEX, Grödig, Austria).

### 2.3. Bacterial Lysis Assay and Time–Kill Assay

Mid-log phase cultures of MRSA T144 (OD_600_ around 0.2) in MHB were treated with PT at different concentrations (0, 8, 16 (1 × MIC), and 32 (2 × MIC) mg/L). A decrease in OD_600_ below the initial value (0.2) indicated cell lysis during the first 4 h.

For standard condition, mid-log bacteria (~10^6^ CFU/mL) were exposed to PT at various concentrations (0, 8, 16, and 32 mg/L) in MHB at 37 °C. For nutrient-deprived condition, bacteria were co-incubated in PBS at 37 °C. For growth arrest condition, bacteria were co-incubated in MHB at 4 °C. The suspensions were withdrawn at 0, 0.5, 1, 2, and 4 h, serially diluted in sterile PBS, and plated on Mueller–Hinton Agar (MHA). Viable colonies were enumerated following 24 h incubation at 37 °C.

### 2.4. MRSA Persisters

MRSA T144 persisters were induced according to a previous study [26]. Briefly, a culture of MRSA T144 grown overnight underwent a 1:100 dilution into 1 mL Luria–Bertani (LB, Land Bridge, Beijing, China) broth until reaching the mid-log phase. Sodium arsenate dibasic heptahydrate (Adamas, Shanghai, China) at 5 mM was subsequently added to the culture for an additional 30 min co-incubation. The induced MRSA persisters were washed with sterile PBS for three times and treated with PT at various concentrations. Vancomycin at 50 mg/L (100 × MIC) was added as the control. Cell viability was assessed using CFU counts during the 4 h exposure treatment.

### 2.5. Effects of Metal Ions on the Anti-MRSA Activity of PT

The metal ions include Zn^2+^, ZnSO_4_; Fe^2+^, FeCl_2_; Mg^2+^, MgSO_4_; Cu^2+^, CuSO_4_; Ca^2+^, CaCl_2_; Mn^2+^, MnSO_4_. Final concentration tested 100 µM for each ion. The effects of metal ions on the antibacterial potency of PT against MRSA T144 were determined in MHB by comparing the OD_600_ of PT with metal ions to the PT control.

### 2.6. Thermostability of PT Against MRSA

PT working solutions were aliquoted into sterile tubes. Solutions were incubated in a temperature-controlled metal bath for 1 h simulating potential storage or pre-treatment conditions at 40 °C (sub-physiological), 50 °C (elevated), or 60 °C (high-temperature stress). The antimicrobial potency of heat-treated PT against MRSA T144 was assessed using plating assays following a 4 h co-incubation period.

### 2.7. Measurement of Total ROS

The intracellular ROS levels in MRSA T144 during treatment were measured using fluorescent 2′-7′-dichlorodihydrofluorescein diacetate (DCFH-DA, 10 μM, Beyotime, Shanghai, China) at excitation/emission wavelengths of 480/530 nm.

### 2.8. Nitric Oxide

Intracellular esterases hydrolyze DAF-2 DA (diamino fluorescein 2-diacetate, Aladdin, Shanghai, China) to DAF-2, which subsequently reacts with nitric oxide (NO) under aerobic conditions to form the highly fluorescent triazole derivative DAF-2 T. Throughout the experiment, NO production in MRSA T144 was quantified by measuring increases in fluorescence intensity (Ex/Em = 485/538 nm).

### 2.9. Lipid Peroxidation Measurement

Lipid peroxidation was determined by C11-BODIPY (10 μM, Bide, Shanghai, China) and Diphenyl-1-pyrenylphosphine (DPPP, 10 μM, Rhawn, Shanghai, China) at excitation/emission wavelengths of 488 nm/540 nm and 351 nm/390 nm, respectively.

### 2.10. ATP Detection

Intracellular and extracellular adenosine triphosphate (ATP) levels were measured using an Enhanced ATP Assay Kit (Beyotime, Shanghai, China). After an hour of co-treatment, the bacterial pellet was lysed to measure intracellular ATP levels. The supernatant was collected to measure the levels of extracellular ATP.

### 2.11. Intracellular pH and Membrane Potential Detection

The intracellular pH (pHi) and membrane potential were determined by 2′,7′-bis-(2-carboxyethyl)-5-(and-6)-carboxyfluorescein, acetoxymethyl ester (BCECF-AM, 5 μM, Beyotime, China) and tetramethylrhodamine ethyl ester (TMRE, 10 μM, TargetMol, Boston, MA, USA), respectively [27].

### 2.12. Membrane Permeability

N-Phenyl-1-naphthylamine (NPN, Macklin, Shanghai, China) and propidium iodide (PI, Macklin, Shanghai, China) were used to measure MRSA membrane permeability at excitation/emission wavelengths of 355/405 nm and 530/590, respectively.

### 2.13. Phospholipid Supplementation Assays

To validate direct interactions between PT and membrane phospholipids, we added PT at 16 mg/L and various phospholipids at 32 mg/L in the MHB culture medium. After 18 h co-incubation with MRSA T144, OD_600_ was recorded. Phospholipids included L-α-phosphatidylglycerol (PG, Aladdin, Shanghai, China; dissolved in DMSO); Cardiolipin (CL, Macklin, Shanghai, China; dissolved in methanol); Phosphatidylethanolamine (PE, Solarbio, Beijing, China; dissolved in ethanol); Phosphatidylcholines (PC, Rhawn, Shanghai, China; dissolved in ethanol); Lysophosphatidylcholines (Lyso-PC, Rhawn, Shanghai, China; dissolved in H_2_O); and Polyene phosphatidylcholine (Polyene-PC, Macklin, Shanghai, China; dissolved in H_2_O).

### 2.14. Analysis of Sliding Motility

Bacterial sliding motility was measured according previous studies [28,29] with modifications. Briefly, 2 μL of the logarithmic solution was spotted onto the semi-solid agar plates (LB 0.4% agar plates supplemented with 0.5% glucose and PT at different concentrations or gentamicin at 8 mg/L). After incubation at 37 °C for 50 h, bacterial motility was determined by measuring the growth zone diameter extending from the inoculation point.

### 2.15. Disinfectant Efficacy Against MRSA on Tableware Surfaces

The tableware was thoroughly cleaned and sterilized before testing. Apply 5 µL of MRSA T144 suspension onto the center of each pre-sterilized tableware material square. Ensure even spreading within a defined area. Once the inoculated droplets have completely air-dried, mimicking contaminated dried residues, PT was applied directly to the dried inoculum spot on each surface square for one hour. The remaining bacterial count was then determined using plate assays.

### 2.16. Biofilm Assays

The inhibitory and eradicative effects of PT on MRSA biofilms were assessed in LB broth supplemented with 1% glucose or 1% milk. For biofilm inhibition assays, PT were added at the same time as inoculum. Following static incubation (37 °C, 24–48 h), biofilms were gently water-washed, stained with 0.1% crystal violet for 15 min, and dissolved in 33% acetic acid for quantification. Absorbance was measured at 615 nm for samples with high biofilm density to ensure readings remained within the linear detection range of the microplate reader. For all other samples, absorbance was measured at 575 nm. For biofilm eradication assays, pre-formed 24 h biofilms were treated with PT for 12 h at 37 °C. The amount of biofilm was also determined by CV assay.

### 2.17. Statistical Analysis

All statistical analyses were performed by Prism 9.0 (GraphPad Software Inc., Boston, MA, USA). All data are presented as mean ± SD. Statistical differences were analyzed by ordinary one-way ANOVA Dunnett’s multiple comparisons among multiple groups. Significance levels were defined as follows: ns (not significant); *, *p* < 0.05; **, *p* < 0.01; ***, *p* < 0.001; and ****, *p* < 0.0001.

## 3. Results

### 3.1. PT Exerts Potent Bactericidal Activity Independent of MRSA Physiological State

**Figure 1:**
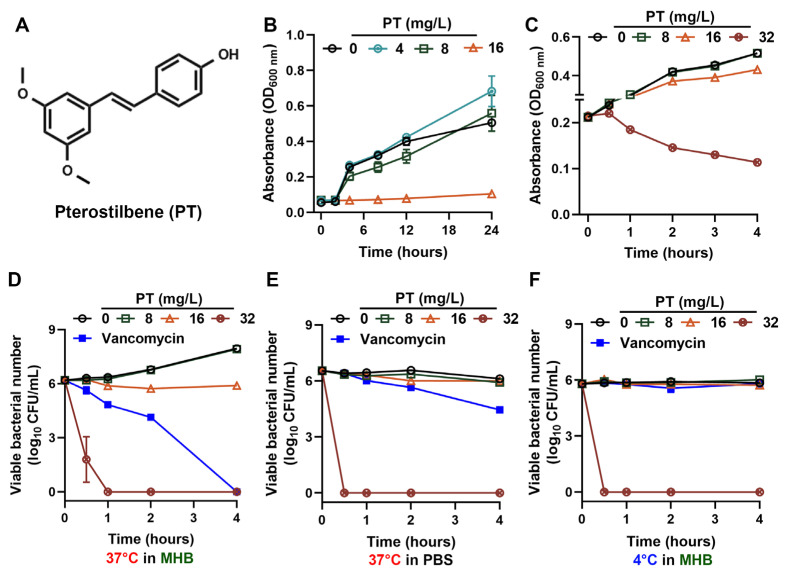
PT kills MRSA independently of physiological state. (**A**) Chemical structure of PT. (**B**) Growth curves (measured by OD_600_) of MRSA T144 collected over a 24 h period to monitor the overall growth inhibition. (**C**) PT induced concentration-dependent lysis in MRSA T144, evidenced by OD_600_ reduction for the first 4 h. (**D**–**F**) The bactericidal killing curves (measured by CFU enumeration) present the results from the first 4 h to highlight the critical early bactericidal effect of PT in different conditions: (**D**) log-phase, MHB at 37 °C, (**E**) nutrient stress, PBS at 37 °C, and (**F**) growth arrest, MHB at 4 °C. Vancomycin at 1 mg/L (2 × MIC) was used as control. Mean values of four replicates were shown, and error bars indicated ± SD.

PT, a dimethylated derivative of resveratrol with the chemical structure depicted in Figure 1A, was demonstrated with superior biocidal activity against MRSA compared to resveratrol [19]. The antibacterial efficacy of PT against the MRSA strains CAU183 and CAU184 was consistent with that against MRSA T144, all exhibiting an MIC of 16 mg/L. Then, MRSA T144 was selected as a representative strain to further investigate the antibacterial properties of PT. As shown in Figure 1B, PT significantly suppressed bacterial growth in a concentration-dependent manner in MHB at 37 °C. At sub-inhibitory concentrations (≤8 mg/L), growth kinetics resembled untreated controls. However, concentrations above 16 mg/L (1 × MIC) induced prolonged lag phases. Moreover, PT induced concentration-dependent bacterial lysis, causing rapid lytic effects in MRSA T144 within 2 h at 32 mg/L (2 × MIC) (Figure 1C). PT at 2 × MIC also eliminated MRSA T144 with equal efficacy across metabolically divergent populations, including logarithmic-phase cells, nutrient compromised cells, and cold stress cells, achieving ≥4-log_10_ CFU/mL reductions within 1 h (Figure 1D–F). As control, vancomycin (2 × MIC) showed state-dependent efficacy, being effective only against MRSA in optimal conditions with MHB at 37 °C. Thus, PT bypasses metabolic limitations, enabling universal killing regardless of bacterial dormancy or stress. Thus, PT circumvents metabolic limitations, thereby achieving comprehensive eradication of MRSA regardless of bacterial dormancy or metabolic stress.

### 3.2. PT Eradicates MRSA Persisters and Maintains Efficacy Across Environmental Conditions

**Figure 2:**
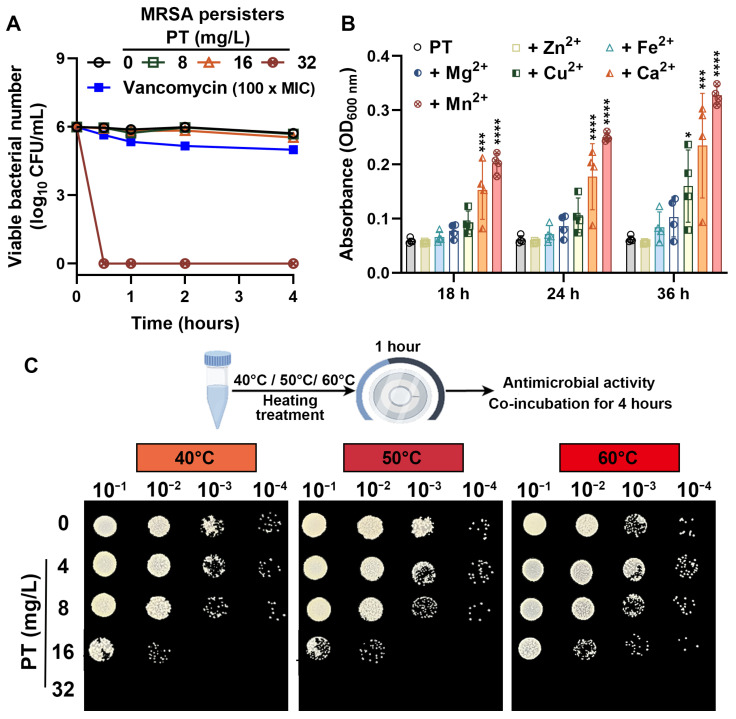
PT rapidly kills MRSA persisters and maintains efficacy across environmental conditions. (**A**) Time–kill kinetics of MRSA persisters treated with PT, or vancomycin (50 µg/mL, 100 × MIC). (**B**) The potent antibacterial activity of PT (16 mg/L) with various metal ions at 100 µM. Data were presented as the mean ± SD of four replicates. Statistical differences were analyzed by ordinary one-way ANOVA Dunnett’s multiple comparisons among multiple groups. Significance levels were defined as follows: *, *p* < 0.05, ***, *p* < 0.001; and ****, *p* < 0.0001. (**C**) Thermostability and antibacterial efficacy of PT after heat treatment at 40 °C, 50 °C, or 60 °C for one hour. Antimicrobial activity of heat-treated PT was determined by plating assays on LB agar plates after 4 h co-incubation.

Given its ability to target metabolically inactive bacteria, we further investigated the potential of PT to eliminate bacterial persisters, a major contributor to chronic and relapsing infections. As expected, persistence populations of MRSA T144 were effectively killed by PT treatment. PT (32 mg/L) achieved >99.9% elimination of viable persisters within one hour, markedly outperforming vancomycin (100 × MIC), which exhibited minimal to no reduction against the same persistence populations (Figure 2A). This indicates that PT’s mechanism of action effectively bypasses the metabolic dormancy that protects bacterial persisters from standard antibiotics. In addition, we determined that certain ions (notably Ca^2+^ and Mn^2+^) reduced the antibacterial activity of PT (16 mg/L) against MRSA T144, potentially by stabilizing target structures or physically blocking PT access. The OD_600_ values for PT + Ca^2+^ and PT + Mn^2+^ were significantly increased from the PT control at 18 h, 24 h, or 36 h (Figure 2B), while the potent antibacterial activity of PT (32 mg/L) was unaffected in the presence of these diverse metal ions at 100 µM. Beyond its resilience to environmental ions, PT also demonstrated significant thermostability. Crucially, PT (32 mg/L) retained potent bactericidal activity against MRSA T144 after heat treatment at 40 °C, 50 °C, or 60 °C for one hour (Figure 2C). Thus, PT’s inherent thermal stability maintains potent antibacterial efficacy under physiologically and environmentally relevant temperature variations.

### 3.3. PT Inhibits Oxidative Stress in MRSA

**Figure 3:**
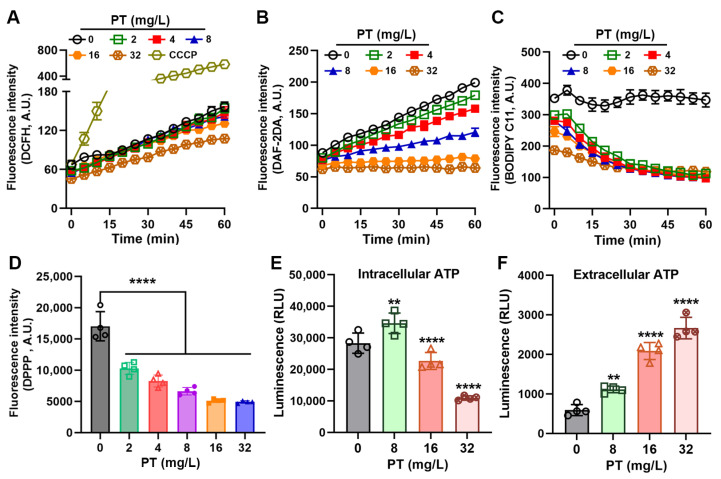
PT inhibits oxidative stress. (**A**) The levels of ROS. (**B**) Intracellular nitric oxide kinetics monitored by DAF-2DA staining. Lipid peroxidation was measured by C11-BODIPY (**C**) and DPPP (**D**). Intracellular (**E**) and extracellular (**F**) ATP contents. RLU means luminescence unit. Mean ± SD (*n* = 4). In (**D**–**F**), statistical differences were analyzed by ordinary one-way ANOVA Dunnett’s multiple comparisons among multiple groups. Significance levels were defined as follows: **, *p* < 0.01; and ****, *p* < 0.0001.

Given the established role of ROS in the killing mechanisms of numerous the bactericidal drugs [30,31], we initially hypothesized that PT might exert its antibacterial effects through ROS generation. The levels of intracellular ROS in MRSA T144 were quantified by DCFH-DA. Surprisingly, PT treatment did not induce ROS accumulation. PT concentration-dependently suppressed ROS production in bacterial cells (Figure 3A). This finding suggested that PT’s antibacterial mechanism operates independently of ROS-dependent pathways. To further characterize its antioxidant activity, we evaluated PT’s impact on nitric oxide (NO) dynamics using DAF-2 DA fluorescence assays. Notably, PT inhibited NO synthesis in a concentration-dependent manner (Figure 3B), reinforcing its capacity to mitigate key oxidative stress mediators. We next examined whether PT attenuated lipid peroxidation using complementary probes BODIPY C11 and DPPP. Accordingly, PT potently inhibited lipid peroxidation across all tested concentrations (Figure 3C,D). This antioxidant effect was particularly pronounced at bactericidal concentrations (≥32 mg/L). Strikingly, ATP dynamics exhibited biphasic pattern: PT at sub-MIC concentration (8 mg/L) induced a transient increase in intracellular ATP, suggesting metabolic stimulation, while PT at above MIC concentrations (16 mg/L or 32 mg/L) decreased the levels of intracellular ATP, which may due to the rapid bactericidal action of PT (Figure 3E). Concurrently, extracellular ATP concentrations exhibited a concentration-dependent elevation upon PT exposure, indicating enhanced membrane permeability (Figure 3F). Collectively, these findings demonstrate that PT’s antibacterial mechanism primarily works through membrane-associated mechanisms rather than oxidative stress.

### 3.4. PT Disrupts Metabolic Gradients and Membrane Integrity

**Figure 4:**
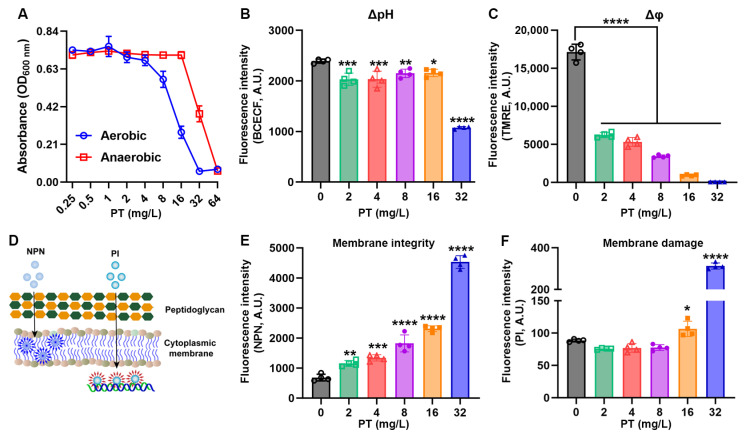
PT disrupts metabolic gradients and membrane integrity. (**A**) PT’s antibacterial efficacy against MRSA T144 in aerobic or anaerobic environments recorded by OD_600_. (**B**) Concentration-dependent cytoplasmic acidification in MRSA T144 measured by BCECF-AM. (**C**) Rapid membrane depolarization quantified by TMRE fluorescence loss. (**D**) Schematic illustration of NPN and PI uptake. (**E**) Membrane integrity determined by NPN uptake. (**F**) Membrane damage determined by PI uptake. Mean ± SD (*n* = 4). In (**B**–**F**), statistical differences were analyzed by ordinary one-way ANOVA Dunnett’s multiple comparisons among multiple groups. Significance levels were defined as follows: *, *p* < 0.05; **, *p* < 0.01; ***, *p* < 0.001; and ****, *p* < 0.0001.

To decouple ROS-dependent effects, we first evaluated PT’s activity under anaerobic conditions. Strikingly, PT’s antibacterial efficacy was significantly attenuated in anaerobic environments, with MIC values increasing 2- to 4-fold (Figure 4A), indicating that oxygen availability modulates—but does not abolish—PT’s lethality. We next probed metabolic disruption by monitoring intracellular pH (pHi) with BCECF-AM. Notably, PT induced a sharp drop in the bacterial pHi at bactericidal concentration (32 mg/L) (Figure 4B). Concurrently, membrane potential (Δφ) collapsed as quantified by TMRE fluorescence decay: 32 mg/L PT caused near-complete depolarization (Figure 4C). This energetic crisis was paralleled by catastrophic membrane damage. NPN uptake assays revealed a concentration-dependent influx of this hydrophobic probe (Figure 4D,E), confirming membrane integrity disruption in MRSA T144. PI penetration assays demonstrated a loss of cytoplasmic membrane integrity in PT treatment at 32 mg/L (Figure 4F). Thus, these data suggest that membrane-targeted disruption by PT may lead to metabolic paralysis.

### 3.5. PT Targets Membrane Phospholipids and Inhibits Biofilm Formation

**Figure 5:**
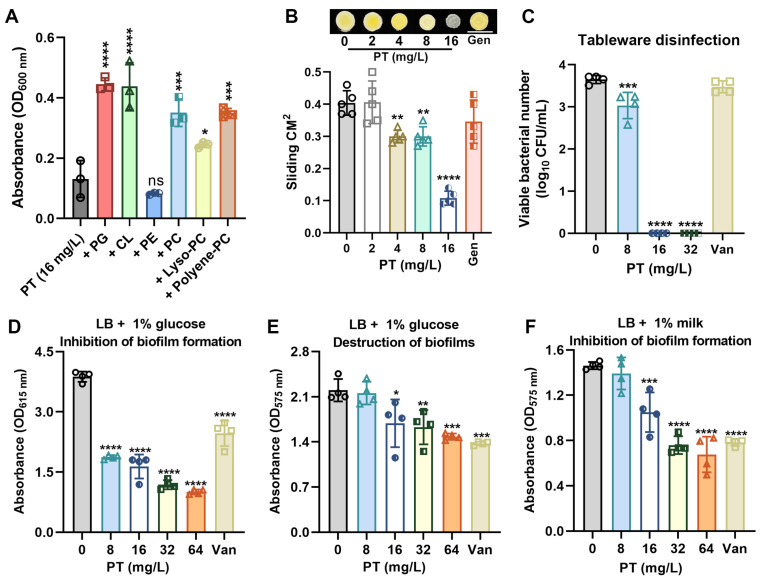
PT interacts with membrane phospholipids and inhibits biofilm formation. (**A**) Phospholipid supplemented with PT against MRSA T144. Mean ± SD (*n* = 3). (**B**) Sliding motility of MRSA T144 on semi-solid agar after 50 h incubation. (**C**) Tableware surface decontamination. (**D**) The inhibitory effects of PT on MRSA biofilms in LB broth plus 1% glucose. (**E**) The eradicative effects of PT on MRSA mature biofilms in LB broth plus 1% glucose. (**F**) The inhibitory effects of PT on MRSA biofilms in LB broth plus 1% milk. Vancomycin at 128 mg/L served as a positive control. In (**B**–**F**), Mean ± SD (*n* = 4). In (**A**–**F**), statistical differences were analyzed by ordinary one-way ANOVA Dunnett’s multiple comparisons among multiple groups. Significance levels were defined as follows: *, *p* < 0.05; **, *p* < 0.01; ***, *p* < 0.001; and ****, *p* < 0.0001.

Building on its established role in membrane permeabilization, we investigated whether PT directly interacts with bacterial phospholipids. When supplemented with exogenous phospholipids including phosphatidylglycerol (PG), cardiolipin (CL) and phosphatidylcholine (PC) at 32 mg/L, the bactericidal activity of PT at 16 mg/L was significantly attenuated (Figure 5A). This competitive reversal confirms PT’s affinity for membrane phospholipids as a primary targeting mechanism. We next assessed PT’s impact on surface motility—a virulence trait dependent on membrane fluidity. Strikingly, PT at sub-MIC (4 mg/L or 8 mg/L) significantly inhibited sliding motility in MRSA T144 (Figure 5B), correlating with altered membrane dynamics, and meanwhile decolorized MRSA colonies (converting staphyloxanthin pigmented colonies to white at 16 mg/L; Figure 5B), indicating disassembled membrane microdomain [32]. Notably, PT exhibited rapid bactericidal action with >3.5-log reduction of MRSA T144 within 1 h, making it suitable for food packaging applications (Figure 5C). Furthermore, PT at sub-MIC (8 mg/L) significantly inhibited biofilm formation (Figure 5D). However, mature biofilms required higher concentrations (above 16 mg/L) for clearance (Figure 5E). The limited eradication efficacy may correlate with poor penetration of PT through exopolysaccharide (EPS) barriers. PT also demonstrated strong biofilm inhibition in the presence of a milk matrix (Figure 5F).

## 4. Discussion

In this study, the antibacterial action of PT, a naturally derived stilbenoid, against MRSA was determined. PT exerts potent lytic activity against MRSA, achieving rapid bactericidal effects independent of bacterial metabolic states, including recalcitrant bacterial persisters. Crucially, PT disrupts key physiological processes: it suppresses ROS generation while paradoxically inhibiting lipid peroxidation, dissipates membrane potential, and inhibits ATP synthesis, all of which may collectively leading to the increased membrane permeability. Notably, the efficacy of PT was significantly attenuated by exogenous phospholipid supplementation. These results indicate that PT’s primary mechanism involves membrane interaction rather than ROS-mediated oxidative damage.

The suppression of basal ROS and lipid peroxidation by PT—contrasting with many membrane disruptors—further confirmed its antioxidant effects. The hydrophobic structure enables direct insertion of PT into the lipid bilayer, causing irreversible membrane disruption and non-ROS-dependent cytotoxicity. The observed partial loss of antibacterial efficacy under anaerobic conditions presents an intriguing aspect of PT’s mechanism of action. This differential activity may be attributed to fundamental changes in the bacterial cell membrane and bioenergetics under anaerobiosis [30,33]. Firstly, anaerobic growth is known to induce significant remodeling of the lipid bilayer in bacteria, often resulting in less rigid and more disordered membranes due to alterations in fatty acid chain composition [34]. The action of amphipathic antimicrobials like PT, which depends on precise insertion into the lipid matrix, could be impeded by such a reduction in membrane packing density and fluidity, thereby reducing its effectiveness. Secondly, the diminished activity may be linked to the crippling of the electron transport chain (ETC) under anaerobic conditions [35], which could reduce the driving force for PT insertion or disrupt its ability to form disruptive pores. However, the ATP surge caused by PT at sub-MIC levels may indicate a bacterial stress response aimed at maintaining homeostasis [36]. PT at bactericidal concentrations overwhelm compensatory mechanisms, causing catastrophic ATP loss. The failed replenishment effort indicates irreversible collapse of energy metabolism. In addition, the massive ATP leakage coincided with the loss of membrane integrity observed in NPN and PI uptake assays and also collectively suggests that membrane disruption—not oxidative stress—is the principal mechanism underlying PT’s bactericidal activity. However, the temporal sequence and causal relationships between membrane permeabilization, ATP production, and ROS modulation are unresolved, warranting real-time kinetic studies employing synchronized bacterial populations.

Within biofilms, a subpopulation of cells can enter a dormant state known as persistence, which confers high levels of tolerance to conventional antibiotics and is a major cause of chronic and recurrent infections [37]. The competitive inhibition by exogenous phospholipids suggests the interaction between PT and membrane components as the initiating event, which may subsequently trigger Δφ collapse and permeability defects, ultimately leading to cell death [38]. Moreover, PT not only prevents biofilm establishment but also effectively disrupts pre-existing, mature MRSA biofilms. The observed efficacy highlights PT’s potential as a promising anti-biofilm agent [39]. The mechanism likely involves interference with cell adhesion, EPS production (potentially through downregulation of genes like *ica* involved in polysaccharide synthesis [40]), and direct bactericidal activity against embedded cells. This dual action of inhibition and eradication positions PT favorably compared to some conventional antibiotics which often show poor penetration and efficacy against biofilms. However, the precise molecular details of PT’s engagement with bacterial membrane lipids remain undefined; this necessitates future biophysical characterization using isothermal titration calorimetry and molecular dynamics simulations to quantify binding affinities.

While this study shows the promising efficacy of PT in a milk model, its application could extend to various other foods. However, stability and sensory effects must be evaluated in each specific matrix. In acidic products such as fruit juices and fermented foods, PT may exhibit greater stability. Nonetheless, potential interactions with ascorbic acid or other organic acids require further study [41]. In high-fat products like meat and processed foods, PT’s lipophilicity may facilitate incorporation into the lipid phase, potentially protecting it from degradation and targeting lipid-rich microbial membranes [42]. However, this could also lead to reduced presence in the aqueous phase where microbes grow, possibly lowering its apparent efficacy. Additionally, the slight phenolic flavor of PT may be masked in strongly flavored products like spiced foods or meat patties but could be detectable in blander systems. Future work should focus on developing delivery strategies (e.g., encapsulation) to improve stability, reduce sensory impact, and ensure effective bioavailability across different food applications.

Importantly, PT is a natural dietary compound found in blueberries and grapes, which provides a foundation for its safety. Human adults can safely consume up to 250 mg of PT per day without damage to vital organs [13]. Studies in mice and rats further demonstrate its safety and anticancer efficacy at doses of 200–500 mg PT/kg body weight, with no significant toxicity observed [16]. The concentrations required for the antimicrobial efficacy demonstrated in our study are considerably lower than these established safety thresholds. Chemical optimization of PT could develop novel analogues with stronger activity against MRSA [43]. Therefore, PT presents a promising and safe candidate for further development as a natural preservative in food systems.

## 5. Conclusions

In conclusion, these findings underscore the multifaceted antibacterial mechanism of PT. PT disrupts membrane integrity and inhibits biofilm formation through interacting with membrane phospholipids. The rapid bactericidal action observed, particularly at 2× MIC concentrations, highlights PT’s potential as an effective antimicrobial agent against MRSA, especially in food packaging applications where rapid clearance is crucial. Notably, PT inhibited biofilm formation even in milk, suggesting strong potential for food industry use. These results advance our understanding of PT’s antibacterial mechanism and lay the groundwork for its therapeutic application.

## Data Availability

The original contributions presented in this study are included in the article material. Further inquiries can be directed to the corresponding authors.
